# Supplementary material for: Smooth Interpolating Curves with Local Control and Monotone Alternating Curvature
Source: Comput Graph Forum. 2022 Oct 6;41(5):25–38. doi: 10.1111/cgf.14600 (PMC9827861; doi:10.1111/cgf.14600)
Supplement: Supplementary file 1 — Supplement Material [file CGF-41-25-s001.zip › Local-Smooth-Interpolating-MonoCurvature/extern/clothoids/submodules/Utils/docs/api-cpp/program_listing_file_rang.cc.html]

Program Listing for File rang.cc — Utils v1.2

### Navigation

- index
- toc
- Utils »
- Program Listing for File rang.cc

# Program Listing for File rang.cc¶

↰ Return to documentation for file (`rang.cc`)

```
#ifndef DOXYGEN_SHOULD_SKIP_THIS

#include "Utils.hh"

#if defined(UTILS_OS_LINUX) || defined(UTILS_OS_OSX)
  #include <unistd.h>
#elif defined(UTILS_OS_WINDOWS)
  #include <windows.h>
  #include <io.h>
  #include <memory>

  // Only defined in windows 10 onwards, redefining in lower windows since it
  // doesn't gets used in lower versions
  // https://docs.microsoft.com/en-us/windows/console/getconsolemode
  #ifndef ENABLE_VIRTUAL_TERMINAL_PROCESSING
  #define ENABLE_VIRTUAL_TERMINAL_PROCESSING 0x0004
  #endif
#endif

namespace rang {

  #ifndef DOXYGEN_SHOULD_SKIP_THIS
  using std::string;
  using std::wstring;
  using std::getenv;
  using std::any_of;
  using std::begin;
  using std::end;
  using std::strstr;
  using std::unique_ptr;
  using std::nothrow;
  using std::clog;
  using std::cerr;
  using std::cout;
  using std::ostream;
  using std::streambuf;
  #endif

  namespace rang_implementation {

    bool
    supportsColor() noexcept {
      #if defined(UTILS_OS_LINUX) || defined(UTILS_OS_OSX)
      static const bool result = [] {
        char const * Terms[] = {
          "ansi",    "color",  "console", "cygwin", "gnome",
          "konsole", "kterm",  "linux",   "msys",   "putty",
          "rxvt",    "screen", "vt100",   "xterm"
        };
        char const *env_p = getenv("TERM");
        if ( env_p == nullptr ) return false;
        return any_of(
          begin(Terms), end(Terms),
          [&](const char *term) { return strstr(env_p, term) != nullptr; }
        );
      }();
      #elif defined(UTILS_OS_WINDOWS)
      // All windows versions support colors through native console methods
      static constexpr bool result = true;
      #else
      static constexpr bool result = false;
      #endif
      return result;
    }

    #ifdef UTILS_OS_WINDOWS

    bool
    isMsysPty( int fd ) noexcept {
      // disable for mingw on MATLAB
      #if (defined(MINGW) || defined(__MINGW32__)) && defined(MATLAB_MEX_FILE )
      return false;
      #else
      // Dynamic load for binary compability with old Windows
      auto const ptrGetFileInformationByHandleEx = reinterpret_cast<decltype(&GetFileInformationByHandleEx)>(
        GetProcAddress(
          GetModuleHandle(TEXT("kernel32.dll")),
          "GetFileInformationByHandleEx"
        )
      );

      if ( !ptrGetFileInformationByHandleEx ) return false;

      HANDLE h = reinterpret_cast<HANDLE>(_get_osfhandle(fd));
      if ( h == INVALID_HANDLE_VALUE ) return false;

      // Check that it's a pipe:
      if ( GetFileType(h) != FILE_TYPE_PIPE ) return false;

      // POD type is binary compatible with FILE_NAME_INFO from WinBase.h
      // It have the same alignment and used to avoid UB in caller code
      struct MY_FILE_NAME_INFO {
        DWORD FileNameLength;
        WCHAR FileName[MAX_PATH];
      };

      auto pNameInfo = unique_ptr<MY_FILE_NAME_INFO>(
        new (nothrow) MY_FILE_NAME_INFO()
      );
      if (!pNameInfo) return false;

      // Check pipe name is template of
      // {"cygwin-","msys-"}XXXXXXXXXXXXXXX-ptyX-XX
      if ( !ptrGetFileInformationByHandleEx( h, FileNameInfo, pNameInfo.get(), sizeof(MY_FILE_NAME_INFO)) ) return false;

      wstring name(pNameInfo->FileName, pNameInfo->FileNameLength / sizeof(WCHAR));

      if ( ( name.find(L"msys-") == wstring::npos && name.find(L"cygwin-") == wstring::npos )
            || name.find(L"-pty") == wstring::npos) return false;

      return true;
      #endif
    }

    #endif

    bool
    isTerminal( streambuf const * osbuf ) noexcept {
      #if defined(UTILS_OS_LINUX) || defined(UTILS_OS_OSX)
      if ( osbuf == cout.rdbuf() ) {
        static const bool cout_term = isatty(fileno(stdout)) != 0;
        return cout_term;
      } else if ( osbuf == cerr.rdbuf() || osbuf == clog.rdbuf() ) {
        static const bool cerr_term = isatty(fileno(stderr)) != 0;
        return cerr_term;
      }
      #elif defined(UTILS_OS_WINDOWS)
      if ( osbuf == cout.rdbuf()) {
        static const bool cout_term = (_isatty(_fileno(stdout)) || isMsysPty(_fileno(stdout)));
        return cout_term;
      } else if (osbuf == cerr.rdbuf() || osbuf == clog.rdbuf()) {
        static const bool cerr_term = (_isatty(_fileno(stderr)) || isMsysPty(_fileno(stderr)));
        return cerr_term;
      }
      #endif
      return false;
    }

    #ifdef UTILS_OS_WINDOWS

    struct SGR {         // Select Graphic Rendition parameters for Windows console
      BYTE    fgColor;   // foreground color (0-15) lower 3 rgb bits + intense bit
      BYTE    bgColor;   // background color (0-15) lower 3 rgb bits + intense bit
      BYTE    bold;      // emulated as FOREGROUND_INTENSITY bit
      BYTE    underline; // emulated as BACKGROUND_INTENSITY bit
      BOOLEAN inverse;   // swap foreground/bold & background/underline
      BOOLEAN conceal;   // set foreground/bold to background/underline
    };

    enum class AttrColor : BYTE {  // Color attributes for console screen buffer
      black   = 0,
      red     = 4,
      green   = 2,
      yellow  = 6,
      blue    = 1,
      magenta = 5,
      cyan    = 3,
      gray    = 7
    };

    HANDLE
    getConsoleHandle( streambuf const * osbuf ) noexcept {
      if ( osbuf == cout.rdbuf() ) {
        static const HANDLE hStdout = GetStdHandle(STD_OUTPUT_HANDLE);
        return hStdout;
      } else if ( osbuf == cerr.rdbuf() || osbuf == clog.rdbuf() ) {
        static const HANDLE hStderr = GetStdHandle(STD_ERROR_HANDLE);
        return hStderr;
      }
      return INVALID_HANDLE_VALUE;
    }

    bool
    setWinTermAnsiColors( streambuf const *osbuf ) noexcept {
      HANDLE h = getConsoleHandle(osbuf);
      if ( h == INVALID_HANDLE_VALUE )  return false;
      DWORD dwMode = 0;
      if ( !GetConsoleMode(h, &dwMode) )  return false;
      dwMode |= ENABLE_VIRTUAL_TERMINAL_PROCESSING;
      if ( !SetConsoleMode(h, dwMode) )  return false;
      return true;
    }

    bool
    supportsAnsi( streambuf const * osbuf ) noexcept {
      if ( osbuf == cout.rdbuf() ) {
        static const bool cout_ansi = (isMsysPty(_fileno(stdout)) || setWinTermAnsiColors(osbuf));
        return cout_ansi;
      } else if ( osbuf == cerr.rdbuf() || osbuf == clog.rdbuf() ) {
        static const bool cerr_ansi = (isMsysPty(_fileno(stderr)) || setWinTermAnsiColors(osbuf));
        return cerr_ansi;
      }
      return false;
    }

    SGR const &
    defaultState() noexcept {
      static const SGR defaultSgr = []() -> SGR {
        CONSOLE_SCREEN_BUFFER_INFO info;
        WORD attrib = FOREGROUND_RED | FOREGROUND_GREEN | FOREGROUND_BLUE;
        if ( GetConsoleScreenBufferInfo(GetStdHandle(STD_OUTPUT_HANDLE),&info) ||
             GetConsoleScreenBufferInfo(GetStdHandle(STD_ERROR_HANDLE),&info) ) {
          attrib = info.wAttributes;
        }
        SGR sgr     = { 0, 0, 0, 0, FALSE, FALSE };
        sgr.fgColor = attrib & 0x0F;
        sgr.bgColor = (attrib & 0xF0) >> 4;
        return sgr;
      }();
      return defaultSgr;
    }

    BYTE
    ansi2attr( BYTE rgb ) noexcept {
      static const AttrColor rev[8] = {
        AttrColor::black,  AttrColor::red,  AttrColor::green,
        AttrColor::yellow, AttrColor::blue, AttrColor::magenta,
        AttrColor::cyan,   AttrColor::gray
      };
      return static_cast<BYTE>(rev[rgb]);
    }

    void
    setWinSGR( rang::bg col, SGR & state ) noexcept {
      if ( col != rang::bg::reset ) {
        state.bgColor = ansi2attr(static_cast<BYTE>(col) - 40);
      } else {
        state.bgColor = defaultState().bgColor;
      }
    }

    void
    setWinSGR( rang::fg col, SGR & state ) noexcept {
      if (col != rang::fg::reset) {
        state.fgColor = ansi2attr(static_cast<BYTE>(col) - 30);
      } else {
        state.fgColor = defaultState().fgColor;
      }
    }

    void
    setWinSGR( rang::bgB col, SGR & state ) noexcept {
      state.bgColor = (BACKGROUND_INTENSITY >> 4) | ansi2attr(static_cast<BYTE>(col) - 100);
    }

    void
    setWinSGR( rang::fgB col, SGR & state ) noexcept {
      state.fgColor = FOREGROUND_INTENSITY | ansi2attr(static_cast<BYTE>(col) - 90);
    }

    void
    setWinSGR( rang::style style, SGR & state ) noexcept {
      switch (style) {
      case rang::style::reset:
        state = defaultState();
        break;
      case rang::style::bold:
        state.bold = FOREGROUND_INTENSITY;
        break;
      case rang::style::underline:
      case rang::style::blink:
        state.underline = BACKGROUND_INTENSITY;
        break;
      case rang::style::reversed:
        state.inverse = TRUE;
        break;
      case rang::style::conceal:
        state.conceal = TRUE;
        break;
      default:
        break;
      }
    }

    SGR &
    current_state() noexcept {
      static SGR state = defaultState();
      return state;
    }

    WORD
    SGR2Attr(const SGR &state) noexcept {
      WORD attrib = 0;
      if ( state.conceal ) {
        if ( state.inverse ) {
          attrib = (state.fgColor << 4) | state.fgColor;
          if ( state.bold ) attrib |= FOREGROUND_INTENSITY | BACKGROUND_INTENSITY;
        } else {
          attrib = (state.bgColor << 4) | state.bgColor;
          if ( state.underline ) attrib |= FOREGROUND_INTENSITY | BACKGROUND_INTENSITY;
        }
      } else if ( state.inverse ) {
        attrib = (state.fgColor << 4) | state.bgColor;
        if ( state.bold      ) attrib |= BACKGROUND_INTENSITY;
        if ( state.underline ) attrib |= FOREGROUND_INTENSITY;
      } else {
        attrib = state.fgColor | (state.bgColor << 4) | state.bold | state.underline;
      }
      return attrib;
    }

    template <typename T>
    inline
    void
    setWinColorNative_tmpl( ostream &os, T const value ) {
      HANDLE const h = getConsoleHandle(os.rdbuf());
      if ( h != INVALID_HANDLE_VALUE ) {
        setWinSGR(value, current_state());
        // Out all buffered text to console with previous settings:
        os.flush();
        SetConsoleTextAttribute(h, SGR2Attr(current_state()));
      }
    }

    #define DO_INSTANTIATION(A)                 \
    void                                        \
    setWinColorNative( ostream &os, A value ) { \
      setWinColorNative_tmpl<A>( os, value );   \
    }

    DO_INSTANTIATION( enum rang::style );
    DO_INSTANTIATION( enum rang::bg );
    DO_INSTANTIATION( enum rang::fg );
    DO_INSTANTIATION( enum rang::bgB );
    DO_INSTANTIATION( enum rang::fgB );

    #endif
  }  // namespace rang_implementation

}  // namespace rang

#endif
```

### Quick search

### Table of Contents

- rang - Colors for your Terminal
- Zstr
- C++ API

«
hide menu

menu
sidebar
»

### Navigation

- index
- toc
- Utils »
- Program Listing for File rang.cc

© Copyright 2021, Enrico Bertolazzi.
Created using Sphinx 4.2.0.
